# Soybean seed proteome rebalancing

**DOI:** 10.3389/fpls.2014.00437

**Published:** 2014-09-03

**Authors:** Eliot M. Herman

**Affiliations:** School of Plant Sciences, BIO5 Institute, University of ArizonaTucson, AZ, USA

**Keywords:** protein, proteome, seed, storage protein, soybean

## Abstract

The soybean seed’s protein content and composition are regulated by both genetics and physiology. Overt seed protein content is specified by the genotype’s genetic framework and is selectable as a breeding trait. Within the genotype-specified protein content phenotype soybeans have the capacity to rebalance protein composition to create differing proteomes. Soybeans possess a relatively standardized proteome, but mutation or targeted engineering can induce large-scale proteome rebalancing. Proteome rebalancing shows that the output traits of seed content and composition result from two major types of regulation: genotype and post-transcriptional control of the proteome composition. Understanding the underlying mechanisms that specifies the seed proteome can enable engineering new phenotypes for the production of a high-quality plant protein source for food, feed, and industrial proteins.

## SOYBEANS ARE A GLOBAL PROTEIN COMMODITY

Among the global commodity of crops, soybean has an almost unique role, being high enough in protein content to provide the nitrogen (N) needed for efficient large-scale animal feed production. Soybeans possess economically valued oil and protein and is an archetype seed used to dissect the processes that specify seed compositional output traits. Over the past decade, considerable public and industry funds have been invested to create soybean community resources, including genomic, transcript, SNP and SSR maps, proteomics, as well as supported a broad range of bioactivity, biochemical, nutritional, and agronomic projects.

## PROTEIN CONTENT AS A GENOTYPE

The genome of soybean specifies the genetic framework for seed formation and maturation, and it controls the expression, mix, and timing for synthesis of the storage metabolite traits (Wilson, 2004 for general information; Hartwig and Kilen, 1991; Wilcox and Shibles, 2001; Chung et al., 2003; Nichols et al., 2006; Bolon et al., 2010). Plant breeding has shown that different soybean genotypes specify a standardized, often line/cultivar-specific protein content (Wilson, 2004). The genetic program that produces seeds is simultaneously manifested in the embryo, endosperm, and maternal plant. Genetic marker analysis, using SNPs, has identified QTLs that demonstrate the overt protein and oil content has a strong genetic determinant (see Diers et al., 1992; Cregan et al., 1999; Zhao-Ming et al., 2011 for examples); these traits have supported generations of breeders who have enhanced soybean as a crop (Brim and Burton, 1979; Carter et al., 1986; Cober and Voldeng, 2000; Wilson, 2004).

Of the three individual components that comprise seeds, two are reproductive progeny: the endosperm and the zygotic embryo, which result from the double fertilization and are enclosed in the maternal-origin seed coat that connects the maturing seed to the maternal plant. Each reproductive-phase soybean plant consists of a coordinated network of embryos and endosperms for the common goal of maximizing reproductive output. Historically, breeding programs have selected traits for enhancement of seed productivity, storage metabolite content, and important agronomic performance traits of the maternal plant. One way to view seed production by plants is as a population that produces (maternal) and distributes (maternal and endosperm) nutrient metabolites to the embryonic sink. During soybean seed development the endosperm undergoes progressive programmed cell death that is completed prior to the accumulation of stored metabolites. By the onset of protein and oil accumulation, only a single cell layer of aleurone remains from the endosperm that encapsulates the embryo, separating it from the inner surface of the maternal seed coat. But the physiological role, if any, of the aleurone in regulating nutrient flux to the developing embryo has not been investigated. Viewed in this way, the soybean plant’s progeny are a population of aleurones and embryos interacting with the metabolite flux. The non-synchronized developing population of seeds must both compete and synchronize with the common maternal nutrient source. To assure the mature seeds are nearly all equivalent in composition, independent of their particular position on the maternal plant, their developmental program and physiological regulation must be coordinated along with the capacity of the maternal plant to nourish them.

The embryo’s genotype specifies maturation-stages (Fehr et al., 1971) that are controlled by transcription factors that provide the developmental framework for storage substance accumulation (Hill and Breidenbach, 1974; Goldberg et al., 1981a,b; Mienke et al., 1981; Walling et al., 1986; Naito et al., 1988; Harada et al., 1989; Perez-Grau and Goldberg, 1989; Nielsen and Nam, 1999; To et al., 2006). A large number of soybean seed-specific DNA binding proteins have been identified (http://casp.rnet.missouri.edu/soydb/), and some of these have been shown to regulate specific seed maturation specific genes. (Chen et al., 1986; Jofuku et al., 1987; Allen et al., 1989; Lessard et al., 1991; Bäumlein et al., 1992; Kwong et al., 2003; Wang et al., 2007). A key role of some transcription factors is to regulate the metabolic and developmental processes that support storage substance accumulation (Kroj et al., 2003; Gutierrez et al., 2007; Santos-Mendoza et al., 2008 for reviews). Understanding how cooperation between the embryo, endosperm, and maternal organs is integrated at the level of gene expression and cross-regulation of metabolism is important for creating models of the source-sink relationship of seed-fill.

## NUTRIENT SOURCE DEFINES SEED PROTEIN ACCUMULATION

Whole plant physiological experiments demonstrate that nutrient distribution to seeds is highly regulated. The seed protein output trait is primarily regulated by controlling the composition of seeds, with the total number of seeds being a consequence of nutrient availability. The average size of seeds from small size plants compared with larger size plants, differs only a little, but the total number of seeds produced is directly related to the total biomass of the maternal plant and its mobilized metabolite source potential. In an agronomic context, this defines yield. From the perspective of the plant, the protein content genotype maximizes the potential of an individual seeds with the overall yield of seeds depending on the available biomass/growth conditions.

Although the maternal plant, endosperm, and embryo function in concert to form the seed, their metabolic interaction occurs without a direct, contiguous flow of nutrients, as each is apoplastically isolated from the other (Thorne, 1980; Thorne and Rainbird, 1983; Egli and Bruening, 2001; Patrick and Oﬄer, 2001 for review). The metabolite flux from the maternal plant through the aleurone to the embryo results from coordinated secretion from the source and uptake by the sink, and this potentially determines the storage output trait (Borisjuk et al., 2003, 2004). In annual plants, such as soybean, the maternal plant must grow rapidly and produce nutrient-capture organs (i.e., roots and leaves).

The relationship between the maternal plant (source) and the seed (sink) has been investigated with increasingly more sophisticated tools and concepts for the past 40 years. For soybean, early studies focused on accumulation of vegetative proteins, primarily in foliage, as the nitrogen store that is mobilized to the seed and determines the accumulation of storage substances. This model of resource acquisition parallels that of most other seed plants, where metabolites in foliage are later mobilized to the seed. The amount of carbon fixed during photosynthesis is highly responsive to the environment, and the maternal plant manages carbon flux so it can distribute nutrients based on their availability and the demand of the seed (endosperm/embryo) sink (see Fellows et al., 1979; Borchers-Zampini et al., 1980 for early examples). A number of studies have shown that leaf proteins, predominantly Rubisco, accumulate over time (Schaefer et al., 1981a,b). In addition, soybean leaves accumulate a vegetative storage protein (VSP), a member of the vacuolar acid phosphatase family (DeWald et al., 1992; Staswick et al., 1994). VSP accumulation is highly responsive to nitrogen availability (Franceschi and Giaquinta, 1983; Franceschi et al., 1983; Staswick, 1989a,b), and it increases with depodding, i.e., removal of the seed sink. This observation led to proposals that VSP is a necessary adjunct that provides additional nitrogen resources for the seed. By silencing the VSP gene, it was later shown that VSP does not appear to make a difference in soybean seed protein content (Staswick et al., 2001).

Accumulated leaf proteins are mobilized by specific proteases (see Ragster and Chrispeels, 1979, 1981a,b) found in leaf cell vacuoles and plastids; the enzymes mediate the hydrolysis of Rubisco and VSP as well as other less abundant leaf proteins. Removal of all maturing seeds, except in one portion of the plant, leads to redistribution of source leaf nutrient flux (Carlson and Brun, 1984) indicating there must be (unknown) feedback-regulation between the seed and the nutrient source that is manifested through a long distance signal. Systems biology approaches could determine how the source size/composition is regulated, and how its mobilization is coordinated with the draw of the sink.

Leaf photosynthate and the products of protein hydrolysis produce a metabolite flux consisting predominantly of sucrose, glutamine and asparagine (Hsu et al., 1984; Rainbird et al., 1984; Krishnan et al., 2011); for nitrogen-fixing legumes in particular, there are also ureides from xylem fluid. With respect to the amino acid flux, the input from glutamine and asparagine has different characteristics. Skokut et al. (1982), using ^15^N-NMR showed that there is no discrimination between the amino and amide N of glutamine, but for Asn the amino N is incorporated into protein twice as efficiently as the amide N, indicating a key role of asparagine in transamination. Asn may also be directly incorporated into proteins, with dual labeled ^13^C and ^15^N Asn being incorporated directly without scrambling the labels (Schaefer et al., 1981a,b). Within the seed, free Asn accounts for a larger fraction of amino acids (33–49%), with the fractional amount varying by genotype (Schaefer et al., 1981a,b). Asn dominates seed coat free amino acid eﬄux, assayed as apoplastic fluid, from the seed cup, i.e., the seed coat with embryo removed (Gifford and Thorne, 1985; Murray, 1987; De Jong et al., 1997; Hernández-Sebastià et al., 2005; Pandurangan et al., 2012). It is unclear whether the tissue source used for these experiments was derived from the inner surface of the aleurone or the maternal seed coat, or a combination of both, since the aleurone often adheres to the inner side of the seed coat. This shows that distinct from the embryo, the seed coat (perhaps comprising aleurone and seed coat) has an amino acid composition similar to the source (assayed as xylem sap), containing Asn as the dominant N-source, and is about 10-fold higher in abundance than Gln (Thorne and Rainbird, 1983; Lohaus et al., 1998; Zhang et al., 2010; Krishnan et al., 2011; Pandurangan et al., 2012).

## PROTEIN COMPOSITION PLASTICITY AND THE SEED PROTEIN CONTENT GENOTYPE

The soybean seed protein output trait has two primary components: total protein content and the composition of individual proteins (the proteome). For soybeans, like many other seeds (Herman and Larkins, 1999 for review), the two major storage proteins, glycinin (11S legumin type) and conglycinin (7S vicilin type), dominate the proteome. The soybean seed proteome also includes many moderately abundant proteins that are bioactive and allergenic, such as the Kunitz and Bowman-Birk trypsin inhibitors, lectin, P34 allergen, sucrose binding protein, urease, oleosins (Herman and Burks, 2011) and several thousand low abundance proteins, including enzymes that mediate metabolism, synthesize storage substances, and create the structural framework of the cell. The specific mix of proteins and each protein’s abundance within the proteome determines the total amino acid composition trait.

Since the development of plant transformation techniques, there have been many attempts to express genes to induce accumulation of large quantities of foreign proteins in seeds. The goals of these projects were often to alter the nutritional quality of seeds, by increasing essential amino acids such as methionine in soybean, or to use the seeds as protein bioreactors. Even with strong storage protein promoters to regulate the transgene expression, the most frequent outcome of such experiments was to produce relatively small amounts of the heterologous protein (about 1% of total) and for the protein and amino acid composition to be little altered compared to control. These minor composition changes occurred whether the protein was targeted to the vacuole or accreted in ER-derived protein bodies, suggesting the limit of protein accumulation is independent of its deposition site. The observed heterologous protein production, compared to the expectations of the engineering design, indicated there must be seed regulatory mechanisms that limit foreign protein production so as not to significantly alter the seed protein content phenotype.

**FIGURE 1:**
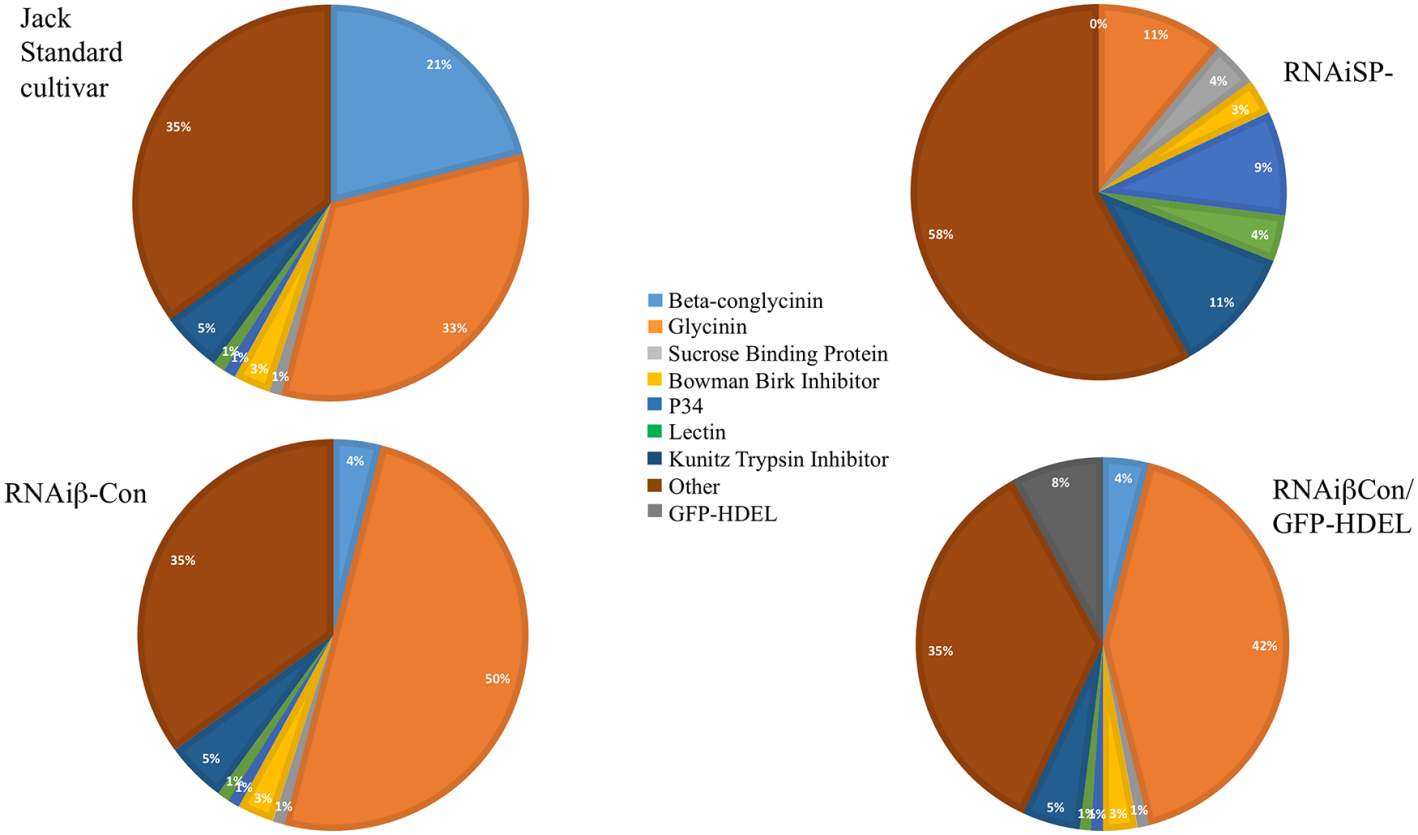
**Variations of seed composition within the context of the protein content genotype are shown.** The pie charts show the relative distribution of proteins comprising the seed proteome of the standard soybean line Jack, a line that silences β-conglycinin α,α′, another line that silences both β-conglycinin and glycinin, and a line where GFP-HDEL forming ER-derived protein bodies mimic of glycinin in the background of β-conglycinin silencing. Each of these seeds contains about the same total protein content while the proteome of each of these seeds varies. This demonstrates how the genomic framework controls the protein content genotype that can be rebalanced by protein composition plasticity.

The converse experiment is to silence the intrinsic major storage protein genes and assess the impact on the seed’s protein content. Kinney et al. (2001) showed total soybean protein content was conserved after silencing conglycinin, which constitutes about 20% of the total seed protein. The resulting seeds accumulated more glycinin, which apparently compensated for the missing conglycinin. Of the five glycinin-genes in the soybean genome, the protein encoded by the glycinin A4 gene tends to accrete in the ER, producing protein (ER)-bodies (Herman and Schmidt, 2004; Herman, 2008), which are not normally found in soybean (Kinney et al., 2001). Mori et al. (2004) showed similar observations in a conglycinin mutant obtained from screening a collection that exhibited the same phenotype of “glycinin rebalancing,” and for some of this protein to remain as proglycinin and to accumulate ER-derived protein bodies. Schmidt and Herman (2008) showed that introducing a gene encoding a foreign protein into the conglycinin-glycinin rebalancing increased the accumulation of the heterologous protein. A GFP-HDEL gene, as a glycinin-gene mimic allele with a glycinin promoter and terminator, was constructed. The addition of the HDEL ER-retention sequence was intended to promote accretion of the protein in the ER to form ER-(protein)-bodies and mimic the accreting glycinin ORF by substituting the GFP-HDEL protein. The expression of GFP-HDEL in the standard cv *Jack* soybean background resulted in about 1% of the total seed protein accumulated as GFP in protein bodies, a level typical of experiments of heterologous protein production in seeds. However, introgressing the GFP-HDEL glycinin mimic allele into the conglycinin-silenced line resulted in about eightfold increased accumulation of GFP as the glycinin mimic was utilized to compensate for the conglycinin shortfall (**Figure 1**).

To further test regulation of the protein content genotype and its capacity to allow for proteome alterations, RNAi silencing of both the glycinin and conglycinin storage proteins (*SP-* or storage protein minus) lines were created that eliminated over two thirds of the protein content of standard soybean seeds (Schmidt et al., 2011). *SP*- seeds exhibited a number of different phenotypes that included conserving the same seed protein content as the parental line, due to compensatory increases of other vacuolar proteins, including Kunitz Trypsin Inhibitor, Lectin, P34, and sucrose binding protein (**Figure 1**). These compensating proteins accumulated at levels up to 11X more than normal, and each protein’s increase occurred without a parallel increase in its steady-state RNA transcript abundance. This suggests that the protein content trait is determined by genotype but the abundance of proteome members occurs at the translational level.

## FREE ASN IS AN INDICATOR OF ALTERED PROTEIN CONTENT AND COMPOSITION

Free Asn is correlated with soybean seed protein content and composition. One line of experimental evidence has shown that in soybean seed cultivars with a range of protein contents there is a positive correlation between high free Asn and high protein content (Hernández-Sebastià et al., 2005; Pandurangan et al., 2012). For the *SP-* storage protein silenced soybean, free Asn increased 5.8X over the standard type (Schmidt et al., 2011). Perhaps in response to the elevated free Asn, the steady-state transcript (RNAseq) level for asparaginase increased 6.5X over the conventional controls. In standard lines the asparaginase level was previously correlated with protein content in standard lines (Wan et al., 2006). Together, these observations suggest there is a correlation of protein increase, whether by genotype selection for higher protein content or by increased abundance of individual proteins within the context of the protein genotype, with changes in free Asn and asparaginase. This suggests the free Asn level is a nitrogen status indicator (Miller et al., 2008), either as a regulator of, or as a component of, the processes that specify protein content and composition.

## CULTURED SOMATIC AND ZYGOTIC EMBRYOS EXHIBIT AN EXCESSIVE GROWTH TRAIT

*Ex vivo* zygotic embryo and somatic embryo cultures are often used as proxies for seed maturation; however, there are significant differences in metabolic behavior of embryos that form *in vivo* and *in vitro* (see Thompson et al., 1977; Obendorf et al., 1983, 1984; Raper et al., 1984; Finer, 1988; Hayati et al., 1996; Santarem et al., 1997; Chanprame et al., 1998; Pipolo et al., 2004; Iyer et al., 2008; Nishizawa and Ishimoto, 2009; Allen and Young, 2013). RNA expression profiling showed that somatic embryos produce a relatively standard set of seed-specific transcripts (Thibaud-Nissen et al., 2003). *Ex vivo* cultures exhibited fidelity with *in planta* seeds, but exhibit differences in the content of accumulated reserve substances (Pipolo et al., 2004). Gln has been shown to be an effective N-input source for these cultures (Saravitz and Raper, 1995; Schmidt et al., 2005) and is often used as the experimental N-source in nutrition-flux studies (He et al., 2011; Allen and Young, 2013; Truong et al., 2013, for recent examples), even though it is Asn that accounts for the large majority of the actual maternal source N *in planta* (Lea and Miflin, 1980; Lohaus et al., 1998; Lima and Sodek, 2003). A recent paper by Allen and Young (2013) showed in cultured zygotic soybean embryos that ^14^C-Gln supplied 36–46% of the carbon of amino acids. In another study using somatic embryos, Truong et al. (2013) showed that increasing Gln in extrinsic culture media resulted in increased protein content, without greater oil content, showing that Gln is preferentially used to synthesize protein. This is consistent with older NMR observations on ^13^C and ^15^N that showed the amino and amido N for Gln as well as the carbon, is non-discriminatory when incorporated into the protein sink (Schaefer et al., 1981b; Skokut et al., 1982).

**FIGURE 2:**
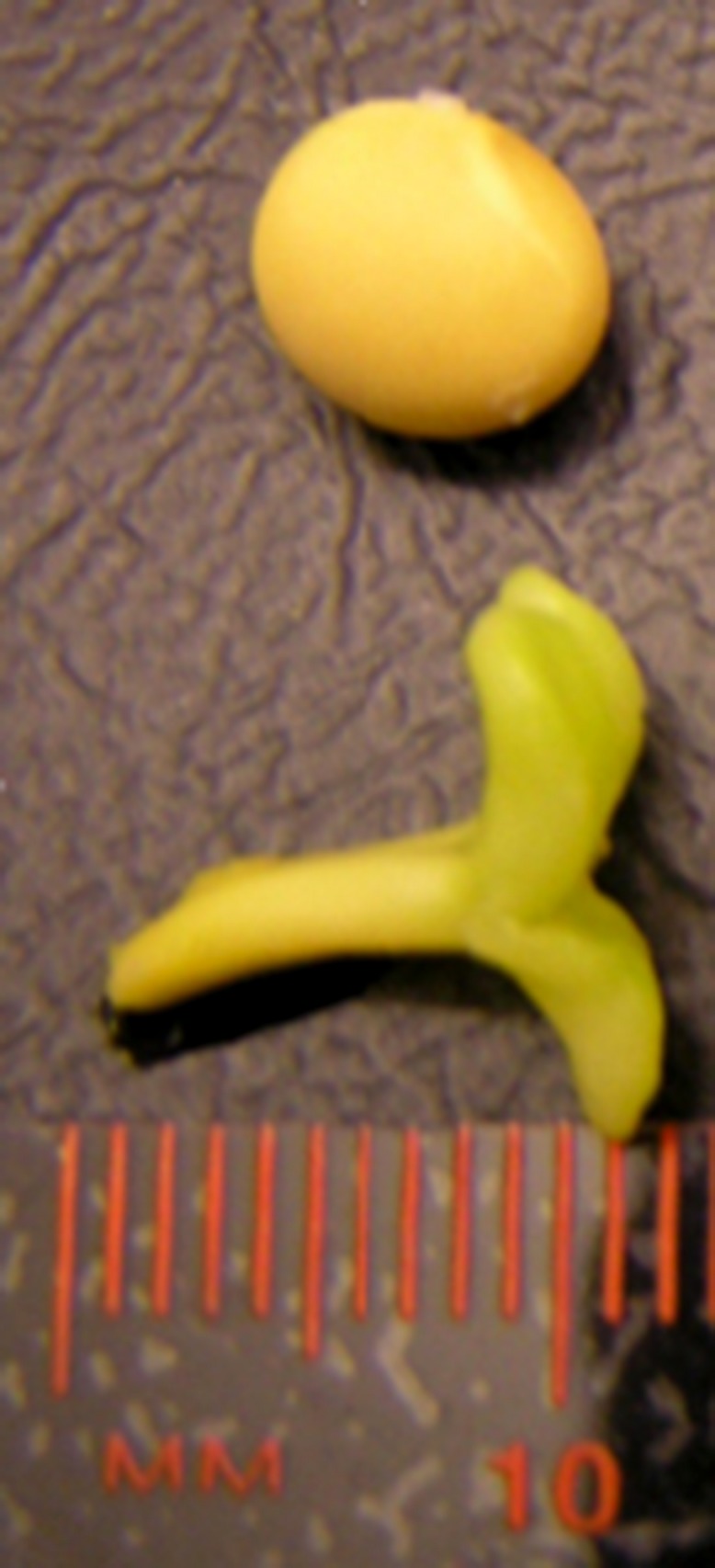
**The comparison of the size and morphology of a mature seed and a mature somatic embryo is shown.** Note that the somatic embryo can exceed the seed’s size, and the axis is enlarged compared to the cotyledon.

**FIGURE 3:**
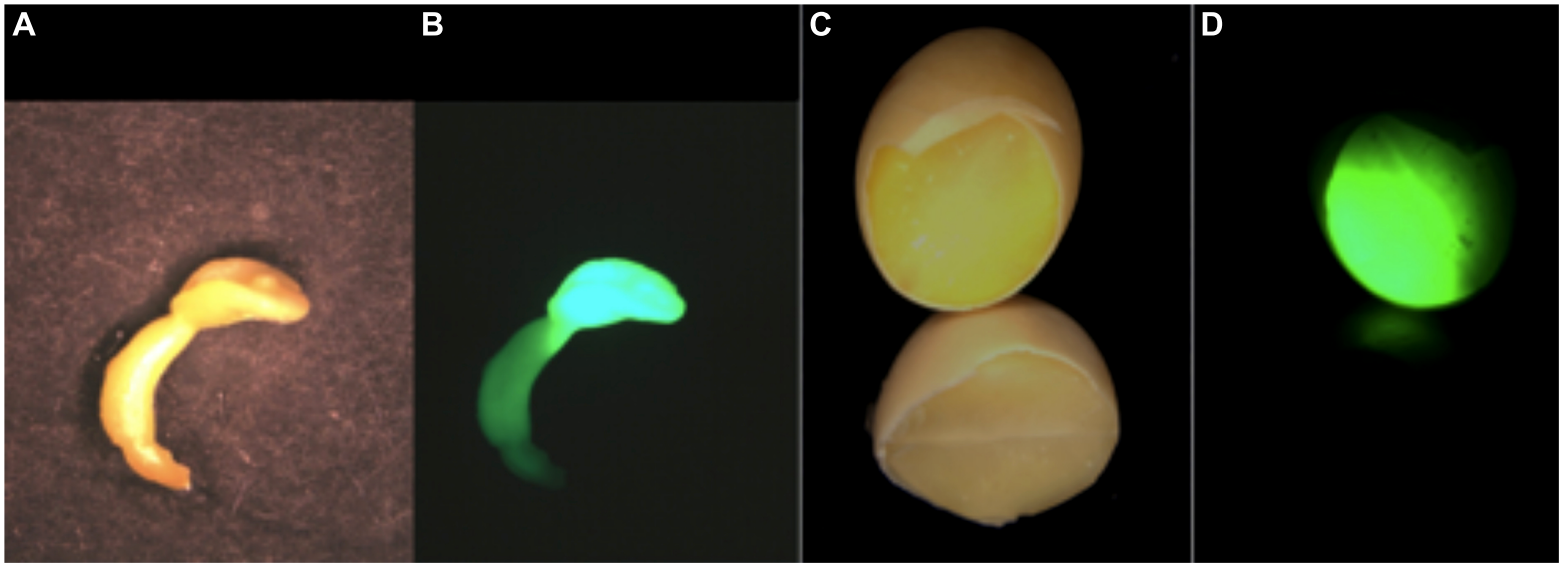
**A comparison of the expression of the storage protein trait in somatic embryos and seeds is shown as a GFP-HDEL glycinin mimic allele expressed as a storage protein proxy.** The somatic embryo in white light **(A)** and UV fluorescence **(B)** shows that the primary GFP expression site is in the cotyledon that is reduced in size compared to cotyledons of seed. In seeds the cotyledons comprise the large majority of the seed’s mass shown as a chipped seed illuminated with white light **(C)** and UV for GFP fluorescence **(D)**. The comparative expression the same glycinin promoter-regulated GFP in somatic embryos and seeds shows that while somatic embryos are a good proxy for seed expression there are challenges in interpreting somatic embryo results as an accurate proxy for a seed’s in planta protein content and composition.

Taken together, these observations support a model where the maternal source supplies Asn (Pandurangan et al., 2012) as the N-source for zygotic embryos, but experimental *ex vivo* embryos can effectively use Gln. The difference between Asn and Gln may be important in the context of the morphological and compositional differences between *in planta* zygotic and cultured somatic and zygotic embryos. The media used for soybean culture varies, although Gln as the N-source dominates (Haga and Sodek, 1987), particularly in SHaM media (Schmidt et al., 2005), which was developed for transgenic embryo maturation (see Schmidt and Herman, 2008; Schmidt et al., 2011 for examples of its use). It is also used in some *ex vivo* nutritional studies (Truong et al., 2013). The media used for immature somatic embryo culture and transformation (Finer, 1988; Finer and McMullen, 1991; Walker and Parrott, 2001) has Asn as the N source. Tissue culture embryos used for transformation and regeneration, freed from the physical and metabolic constraints of the endosperm/aleurone and seed coat, exhibit aberrant growth (**Figures 2** and **3**), supporting a regulatory role for the endosperm and/or seed coat in seed development (Garcia et al., 2003, 2005; Berger et al., 2006). In culture, somatic embryos form “monster” embryos with an enlarged embryonic axis and diminished, sometimes fused, cotyledons. Somatic embryos grown in the SHaM media are deemed “healthy,” (i.e., large, green, well-formed), often exceeding in size a fully formed seed. Similar observations are obtained by culturing immature zygotic cotyledons that enlarge to a size that exceeds that of *in planta* seed cotyledons. This suggests the more an embryo is fed, the larger it grows, even beyond the size in a standard seed. Cultured embryos favor the accumulation of carbonaceous over nitrogenous metabolites, yielding less protein per mass than zygotic embryos. For *ex vivo* zygotic and somatic embryos, the genotype-specific protein content and its proteome phenotype appears to be less regulated, and instead the storage substance accumulation appears to have a direct relationship with nutrient input. The differences between *in planta* and *ex vivo* embryo development and storage substance accumulation indicates the significance of the *in planta* circumstance of each seed as an interactive member of a larger population.

## SEED PROTEIN CONTENT AND ITS VARYING PROTEOME

For seed crops, of which soybean is a prominent example, historic and modern breeding has selected for enhanced storage substance accumulation. Generations of breeders have established that protein content is a genetically determined trait that can be selected. How protein content is regulated in relationship with protein composition appears to be a multilevel process, with the genotype establishing protein content. The rebalancing of proteome occurs in both dicots and monocots as shown by observations on soybean and maize (Wu and Messing, 2014). From the perspective of the seed that has to establish the next generation of an annual plant, its capacity to make compositional choices in response to altered metabolic circumstances has selective advantages. Understanding the processes that control proteome plasticity within the context of the protein content phenotype can enable biotechnologists to create enhanced soybeans optimized for specific end uses, such as species-specific feed or as protein bioreactors.

## Conflict of Interest Statement

The author declares that the research was conducted in the absence of any commercial or financial relationships that could be construed as a potential conflict of interest.
